# A Guide RNA Sequence Design Platform for the CRISPR/Cas9 System for Model Organism Genomes

**DOI:** 10.1155/2013/270805

**Published:** 2013-10-03

**Authors:** Ming Ma, Adam Y. Ye, Weiguo Zheng, Lei Kong

**Affiliations:** ^1^Biomedical Engineering Department, College of Engineering, Peking University, Beijing 100871, China; ^2^Center for Bioinformatics, State Key Laboratory of Protein and Plant Gene Research, School of Life Sciences, Peking University, Beijing 100871, China; ^3^National Institute of Biological Sciences, 7 Science Park Road, Zhongguancun Life Science Park, Beijing 102206, China; ^4^Institute of Computer Science and Technology, Peking University, Beijing 100871, China

## Abstract

Cas9/CRISPR has been reported to efficiently induce targeted gene disruption and homologous recombination in both prokaryotic and eukaryotic cells. Thus, we developed a Guide RNA Sequence Design Platform for the Cas9/CRISPR silencing system for model organisms. The platform is easy to use for gRNA design with input query sequences. It finds potential targets by PAM and ranks them according to factors including uniqueness, SNP, RNA secondary structure, and AT content. The platform allows users to upload and share their experimental results. In addition, most guide RNA sequences from published papers have been put into our database.

## 1. Introduction

Gene engineering technology has always been a hot topic in life science research. With the development of gene modification technology, certain genes can be knocked out or knocked down to a lower level. The appearance of zinc finger nuclease (ZFN) and tale nuclease (TALEN) has greatly accelerated progress in this field, but their efficiency is often unpredictable and it is difficult to target selected genes [1–8].

Recently, Cas9/CRISPR has been reported to successfully induce targeted gene disruption and homologous recombination in both prokaryotic and eukaryotic cells with higher efficiency compared with ZFN and TALEN [9–13]. Additionally, it is easier to design guide sequence and easy to use for Cas9/CRISPR system [10]. This novel technology will be of great potential for application in both research field and clinical trials.

However, there is no available tool for the guide RNA design of Cas9/CRISPR silencing system. Although Mali et al. have reported the construction of unique whole human genome guide RNA library, covering more than 40% human exons [9], they did not provide a tool for researchers to design novel target sequences for other model organisms.

Existed library also did not take into consideration related influencing factors, such as SNP, deletion or insertion on the genome, and potential RNA secondary structure. According to our current understanding of the gRNA maturing process, the secondary structure of gRNA is crucial for Cas9-gRNA complex [14]. The 20 bp guide RNA sequence is used to bind with target site in genomes. If they are mostly involved into RNA loops, the efficiency to bind with target sites would be low. Thus, this factor should be taken into consideration. Besides, the interference efficiency is probably closely related to the melting temperature of the gRNA-DNA hybrid. A relatively high AT content is negatively correlated with the off-target effect, and thus sequence with extremely low AT percentage is, to some extent, not recommended [9].

Thus, we developed an online platform for the guide RNA design of the Cas9/CRISPR silencing system (http://cas9.cbi.pku.edu.cn/), with DNA variants information integrated. This tool helps researchers design their candidate guide RNA sequences more easily and provides assistance for users to choose better candidates based on preliminary results.

## 2. Materials and Methods

Both guide RNA sequences and their corresponding efficiency were manually collected from the literature and stored in our database. For designing guide RNA, we used a Java framework mainly containing 5 steps, and connecting to Tomcat web server. 

In the first step, the program would find any candidate sequences based on the N_20_NGG sequence pattern principle, where NGG represents PAM sequence, by utilizing Java regular expression matching. In the second step, the program would put all the candidate sequences to a fasta file and run bowtie 0.12.9 to check if they could be mapped on selected model organism's genome uniquely [15]. The parameters for bowtie were “-f -v 1 -k 10 -l 16 –S,” as “-f” told bowtie the input was fasta file, “-v 1” for only allowing at most one mismatch, “-k 10” reporting up to 10 good alignments, “-l 16” setting seed length to 16, and “-S” outputting sam format. As the length of target region was only 23 bp, the default seed length 28 for bowtie was not proper for this job, so we adjusted it to 16. We thought the number of mismatches might largely affect effectiveness, and this step mainly focused on checking the mapping uniqueness, so we just looked for hits with at most one mismatch and output at most 10 hits. The mapping result would be parsed in Java, and then, in the third step, would call tabix 0.2.5 to find out any overlapped SNPs or indels as reported in dbSNP135 [16–18], if the target genome was human hg19. The dbSNP135 vcf file was downloaded from GATK bundle. In the fourth step, it would predict RNA secondary structures for those candidate gRNA sequences by calling Vienna RNAfold 2.0.7 with default parameters [19]. In the last step, the program rearranged all the information for the designed gRNA and formatted it to better-looking HTML. The AT% and the distance of the variants to the 3′ end of the target region were also calculated. The output gRNAs were sorted by both number of mapping hits and number of overlapping SNPs. The time consumption for this pipeline was mainly on running bowtie and sometimes tabix, when there existed many target sequences, and was roughly about three seconds for one query sequence.

## 3. Results and Discussion

**Figure 1:**
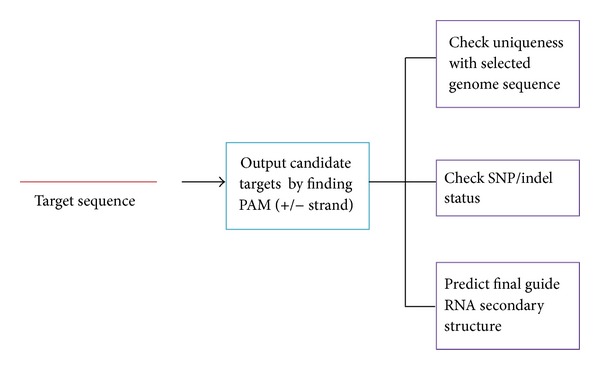
Streamline of guide RNA design platform. Target sequences are searched for the whole genome for uniqueness, and then check SNP/indel status. The results are output from top to bottom with more unique and fewer SNP/indel. The entire gRNA secondary structure is also given as reference.

**Figure 2:**
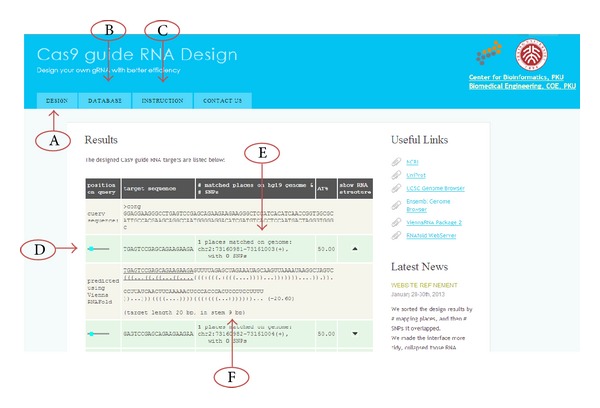
Instruction of platform function. Overview of platform interface. (A)–(C) represent functions and database. (D) represents sense/antisense and position information of output sequences on target sequences. (E) represents uniqueness and SNP/indel status. (F) represents mature gRNA secondary structure.

Multiple gene sequences are allowed for batch gRNA design and the streamline of this platform is shown in Figure 1. The results contain genomic loci information of gRNAs and SNP/INDEL inside them. This would help researchers choose a more unique target candidate and avoid SNP/insertion/deletion. Moreover, this platform evaluates all candidates based on their RNA secondary structure and AT content, allowing users to choose better candidates (Figure 2).

Recently, Jiang et al. report that only the first six base pairs near PAM are of great importance for recognition efficiency in bacteria [20]. It is unknown whether or not this is still the case for eukaryotic or even mammalian cells. We will keep updating our algorithm to rank candidate gRNAs.

We conducted a validation by using those reported results in our platform on factors, such as uniqueness, SNP, and base in loops (Table 1, italic font represents low efficient targets). The more unique, with fewer SNPs and base in loops, generally the gRNA is more efficient. For the given gene *PVALB*, the first target sequence is 50% more efficient than the rest two, since the first has 0 SNP while the rest have 3 or 2 SNPs. The first target sequence has fewer base pairs involved in RNA secondary structure loops, allowing it to bind more with target genome, while the rest two both have 9 base pairs in loops. For the given gene *AAVS1*, the first target is more than twofold efficient than the other, since the other one has an off-target site in genomes. For the given gene *VEGFA*, the first one is about half efficient with the rest two, since it has 1 SNP while the rest have none.

AT content is crucial factor as those previously mentioned, since evidence is not clear. Thus, we list it here as a consideration for users.

## 4. Conclusions

Our platform is an easy-to-use software to identify potential efficient gRNA sites within given sequences for model organisms, avoiding off-target effects and SNPs. This platform also allows users to search existing guide RNA/protospacer sequences and share their results. We have manually extracted most reported gRNA/protospacer sequences into our database for reference and will expand it with newly published work.

## Figures and Tables

**Table 1:** Analyze of reported targets in human cells in this platform.

Target genes	Guide RNA sequences	Mapping and SNP	bp in loops	AT%	Efficiency	Methods	Reference
Human **PVALB**	ATTGGGTGTTCAGGGCAGAG	1 places matched on genome: chr22:37196884-37196906(+), with 1 SNPs: rs12483924 (2 bp to 3′ end)	6	45%	6.50%	Surveyor	Cong et al. 2013 [10]
*Human * ***PVALB***	*GTGGCGAGAGGGGCCGAGAT *	*1 places matched on genome: chr22:37196866-37196888(+), with 3 SNPs: rs3484 (18 bp to 3*′* end) rs181855770 (10 bp to 3*′* end) rs9607383 (9 bp to 3*′* end) *	*9 *	*30% *	*ND *		
*Human * ***PVALB***	*GGGGCCGAGATTGGGTGTTC *	*1 places matched on genome: chr22:37196875-37196897(+), with 2 SNPs: rs181855770 (19 bp to 3*′* end) rs9607383 (18 bp to 3*′* end) *	*9 *	*35% *	*ND *		

Human **AAVS1**	GGGGCCACTAGGGACAGGAT	1 places matched on genome: chr19:55627117-55627139(−), with 0 SNPs	8	35%	8.07%	HR	Mali et al. 2013 [9]
*Human * ***AAVS1***	*GTCCCCTCCACCCCACAGTG *	*2 places matched on genome:chr19:55627136-55627158(−), with 0 SNP schr4:108975634-108975656(+), with 1 SNPs: rs115503552 (7 bp to 3*′* end) *	*7 *	*30% *	*3.26% *		

*Human * ***VEGFA***	*GGGTGGGGGGAGTTTGCTCC *	*1 places matched on genome: chr6:43737291-43737313(−), with 1 SNPs: rs12210204 (1 bp to 3*′* end) *	*11 *	*30 *	*26% *	T7EI assay	Fu et al. 2013 [21]
Human **VEGFA**	GACCCCCTCCACCCCGCCTC	1 places matched on genome: chr6:43738556-43738578(−), with 0 SNPs	4	20	50%		
Human **VEGFA**	GGTGAGTGAGTGTGTGCGTG	1 places matched on genome: chr6:43737454-43737476(+), with 0 SNPs	12	40	49.40%		

*ND represents not detectable. Italic font represents low efficient gRNAs within the same gene group.
